# Supplementary material for: Multicentric study on the reproducibility and robustness of PET-based radiomics features with a realistic activity painting phantom
Source: PLoS One. 2024 Oct 24;19(10):e0309540. doi: 10.1371/journal.pone.0309540 (PMC11500893; doi:10.1371/journal.pone.0309540)
Supplement: Attachment — Submitted filename: PONE-D-24-08717_review.pdf [file pone.0309540.s002.pdf]

The authors of this manuscript focus on investigating a multicentric study on the reproducibility and robustness of PET-based radiomics features with a realistic activity painting phantom. As they state, there exist certain variability on the obtention of PET radiomic features, as well as in the associated radiomic pipelines. Working towards harmonization of PET images and homogenization of the pipelines is desirable, but there still exist different factors hindering the establishment of reproducible and robust PET radiomic pipelines. Their methods and findings underscore the need for such standardization in PET radiomics.

The topic of the manuscript is of interest in the community; however, the manuscript frequently lacks clarity and is difficult to follow. Considering a few points would improve the overall quality of the manuscript and improve its content:

The ideas presented in the introduction seem a bit isolated and, sometimes, it is difficult to read and follow, with the connection between paragraphs is a bit weak; I would suggest the authors to revisit the introduction, extend it, and improve its overall flow.

The authors should be more careful when reporting data. For example, in table 1, there are values reported with different resolutions (i.e., volume, or time inaccuracy). Please check for consistency.

Figure 2 should describe the actual orthogonal orientations of the images.

The authors write several times about the measurements being carried out with the application of the PET scanners. This is a bit weird to say, as, even if PET counts are measured, images are acquired, or better said, data is acquired and images reconstructed, using the PET scanners.

In table 2, the authors should better describe the contents of the different cells. Not everyone reading the paper should be able to understand the reconstruction settings, regarding algorithms (OSEM, TT3D, etc.) and their parameters (i.e., iterations, subsets, etc.). Table caption should, at least, include the definition of the acronyms and the format of number of iterations and number of subsets.

What is a “standard” reconstruction here?

Codes C, D, E and F are reported with no filtering. In which scenarios is data not filtered and smoothed? Most clinical data are smoothed in some way, so I am wondering how close to the reality those post-processing steps are.

It is unclear how the authors calculated the radiomic features. In-house? Any package?

The authors compute first order statistics, neglecting the description of histogram and intensity radiomic features such as skewness, kurtosis, energy, etc. which are commonly used in radiomic approaches, but then are included in figure 4. They should better describe how they actually compute the radiomic features and which ones they are including in the study.

Apart from those and high order features, the authors may want to discuss the use of transformations and radiomic features derived from those transformations.

Also, the nature of the phantom and the whole work neglect the inclusion of the shape features, which are agnostic and more robust than the rest of features… But again, shape features are included in figure 4 (despite being just a few). This should be better described, noted and discussed by the authors.

In the statistical analysis, the authors chose the D imaging setting arbitrarily; however, I am not sure if that was a good decision, as that option uses the “standard” unknown method and has no post-processing, which implied it might be more contaminated by noise.

Figure 3 lesions do not seem to match lesions from figure 2. Please explain and include the lesions from figure 2 as a main row at the top of figure 3. Also, include a colorbar showing the actual values of the PET uptake (are those just MBq or SUV?).

Figure 4 does not only show relative, but also absolute, differences in features. This seems to be inconsistent with the text. Also, why there is no 0 value? If two methods are equivalent, that value should be considered… Additionally, relative values could be negative. These results should be revisited and better reported/discussed.

Figure 6 shows the same trend and potential error, as all abs and rel values are the same! Are these values correct or is the error always biased on the same direction?

How was the Wilcoxon signed-rank test run? Did the authors consider the number of tests and correct for multiple comparisons? How? Given the great number of tests, there could be some statistical results that are just derived from random results.

Conclusions should include a better summary of the contribution to increase the overall soundness.
